# Xanthogranulomatous pyelonephritis complicating sepsis and abdominal compartment syndrome

**DOI:** 10.1097/MS9.0000000000004975

**Published:** 2026-04-29

**Authors:** Jialong Zheng, Yijie Chen, Chenghua Zhang, Jianshe Shi

**Affiliations:** Department of Surgical Intensive Care Unit, Huaqiao University Affiliated Strait Hospital, Quanzhou, China

**Keywords:** abdominal compartment syndrome, case report, multidisciplinary care, nephrectomy, sepsis, XGP

## Abstract

**Introduction and Importance:**

Xanthogranulomatous pyelonephritis (XGP) is a rare chronic kidney infection characterized by tissue destruction and lipid-laden macrophage infiltration. While sepsis is a known complication, abdominal compartment syndrome (ACS) is extremely uncommon. This case underscores the importance of early recognition and timely surgical intervention in managing life-threatening sequelae of XGP.

**Presentation of Case:**

A 43-year-old woman with a history of substance abuse and recurrent urinary tract infections presented with septic shock and abdominal distension. Imaging showed classic signs of XGP with mass effect. Initial treatment included antibiotics and nephrostomy drainage. She later underwent a left radical nephrectomy. Histopathology confirmed XGP without calculi, raising the possibility of a methamphetamine-related immune mechanism. The patient recovered fully with preserved renal function.

**Clinical Discussion:**

XGP is frequently misdiagnosed due to its nonspecific clinical and radiological features. When complicated by sepsis and intra-abdominal hypertension, the condition can rapidly progress to multiorgan failure. This case highlights the need for high clinical suspicion, multidisciplinary collaboration, and prompt surgical management in severe presentations of XGP.

**Conclusion:**

This rare case of XGP complicated by ACS highlights the importance of early diagnosis, timely drainage, and multidisciplinary care. A non-lithogenic origin should be considered in atypical cases, especially in patients with a substance use history.

## Introduction

Xanthogranulomatous pyelonephritis (XGP) is a rare and severe kidney condition where kidney tissue is destroyed and lipid-laden macrophages (foam cells) accumulate. XGP accounts for about 1% of chronic pyelonephritis cases, with an annual incidence of 0.6 to 1.4 cases per 100 000 people^[^1^]^. It mainly affects middle-aged women, especially those with recurrent urinary tract infections (UTIs), diabetes, or immune system problems. XGP is often linked to chronic UTIs, blockages in the urinary system, and kidney stones, though some rare cases occur without stones, suggesting other causes like immune issues or substance abuse.

XGP typically presents with symptoms such as fever, flank pain, and general discomfort, but it can lead to life-threatening complications, including sepsis, kidney failure, and abdominal compartment syndrome (ACS). Although XGP is not uncommon in chronic pyelonephritis, the development of severe complications like ACS is very rare.

HIGHLIGHTS

- This case highlights a rare and severe complication of xanthogranulomatous pyelonephritis (XGP) in a young patient.
- The patient developed septic shock and abdominal compartment syndrome in the setting of XGP.
- Early recognition and prompt surgical intervention played a critical role in the patient’s survival.
- This case emphasizes the importance of considering XGP in patients with obstructive uropathy and systemic signs of infection.
- To our knowledge, this represents one of the few reported cases of XGP complicated by both sepsis and abdominal compartment syndrome in the English literature.

This report describes a case of septic shock and ACS caused by XGP in a patient with a history of substance abuse. The aims of this study were: (1) to review the clinical features of this patient, diagnostic imaging, and initial antibiotic treatment; (2) to summarize the literature on XGP-related sepsis, ACS risk factors in XGP, and severe XGP management strategies; (3) to provide evidence for multidisciplinary management and initial antibiotic choices for critically ill patients with septic shock caused by XGP. This case report has been reported in line with the SCARE checklist.

## Case presentation

**Figure 1.**
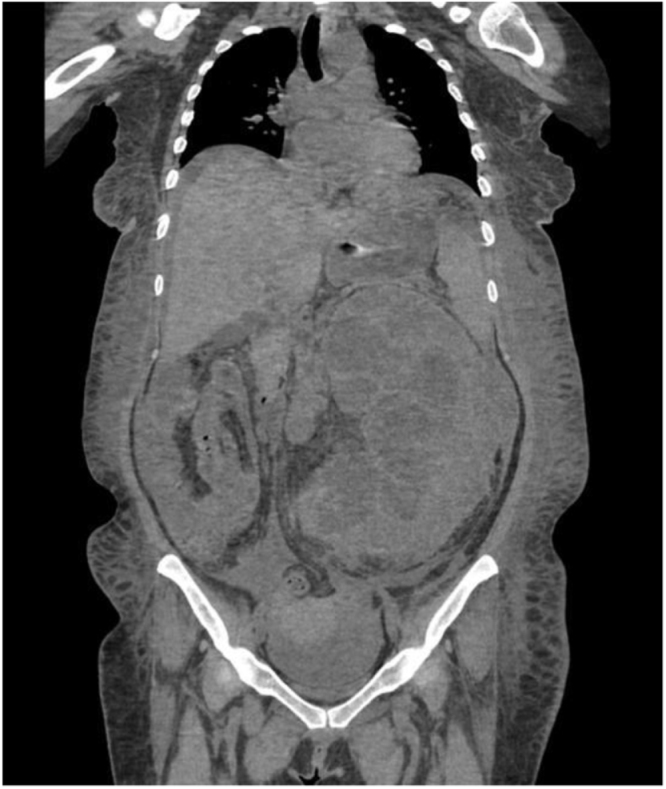
Abdominal computed tomography showed that the left kidney was significantly enlarged and presented as the “bear’s paw sign”.

**Figure 2.**
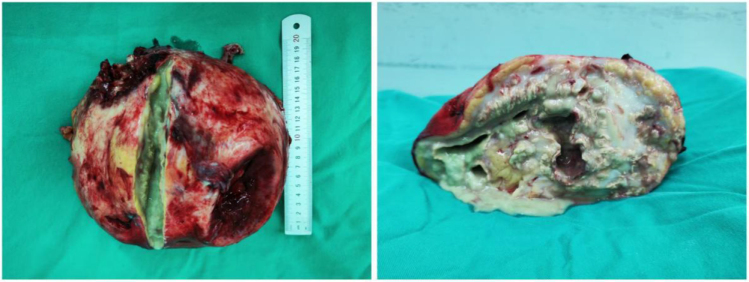
The surgically removed left kidney, upon being incised, revealed a large amount of pus and necrotic tissue.

A 43-year-old woman arrived at the emergency department in severe distress, with tachycardia, rapid breathing, and a swollen abdomen. Her medical history was notable for substance abuse and recurrent UTIs. Her body mass index was not available at the time of admission. Lab results showed a white blood cell count of 37 270 per cubic millimeter (reference range, 4500 to 10 000), a serum creatinine level of 327.3 µmol/L (reference range, 53 to 133 µmol/L), and a serum lactate level of 3.7 mmol/L (reference range, 0.5 to 2.2 mmol/L). Urinalysis revealed 20 white blood cells per high-powered field. Bacterial cultures obtained from both urine and nephrostomy drainage fluid identified *Proteus mirabilis*. Antimicrobial susceptibility testing was performed using minimum inhibitory concentration (MIC) determination and the Kirby–Bauer disk diffusion method, with results interpreted according to Clinical and Laboratory Standards Institute (CLSI) guidelines. The Sequential Organ Failure Assessment (SOFA) score was 8, and intravesical pressure was 21 mmHg. Computed tomography (CT) scans showed a notably enlarged left kidney with cortical atrophy and calyceal dilatation, with a “bear paw sign” (Fig. 1). This anatomical alteration exerted a notable mass effect, compressing adjacent visceral structures, displacing the diaphragm cephalad (thereby inducing the “baby lung” appearance), and contributing to ascites formation. A diagnosis of sepsis and ACS due to XGP was confirmed. Initial treatment included aggressive fluid resuscitation and broad-spectrum antibiotics (cefoperazone and sulbactam sodium). Ultrasound-guided nephrostomy drained thick, beige purulent material. *Proteus mirabilis* was isolated from both the nephrostomy drainage and urine cultures (Table 1). After initial stabilization, the patient underwent a left radical nephrectomy (Fig. 2). Gross examination revealed marked hydronephrosis with dilated calyces and multiloculated cystic cavities filled with yellowish necrotic material. Histopathological analysis revealed extensive fibrosis and chronic granulomatous inflammation, characterized by lipid-laden foamy macrophages (CD68 +), mixed plasmacyte infiltrates (CD138 +/CD38 +), and chronic lymphadenitis. This pathologically confirmed case of non-lithogenic XGP suggests a potential methamphetamine-induced injury mechanism, mediated by T-cell dysfunction. The patient was weaned off mechanical ventilation by postoperative day 12 and was discharged on day 36 with preserved renal function at the 3-month follow-up.

**Table 1:** Antibiotic susceptibility of *Proteus mirabilis* in drainage fluid culture from nephrostomy.

Antibiotic	Test method	Result (µg/ml or mm)	Susceptibility
Piperacillin/Tazobactam	MIC	≤4	S
Cefoperazone/Sulbactam	MIC	≤8	S
Ceftriaxone	MIC	≤0.25	S
Ceftizoxime	MIC	≤0.12	S
Cefuroxime Axetil	MIC	≤1	S
Imipenem	KB	23 mm	S
Meropenem	KB	27 mm	S
Levofloxacin	MIC	0.5	S
Amoxicillin/Clavulanic Acid	MIC	≤2	S
Cefuroxime	MIC	≤1	S
Ceftazidime	MIC	≤0.12	S
Cefoxitin	MIC	≤4	S
Aztreonam	KB	34 mm	S
Ertapenem	MIC	≤0.12	S
Amikacin	MIC	≤2	S
Trimethoprim/Sulfamethoxazole	MIC	≤1	S

MIC, Minimum Inhibitory Concentration (μg/ml); KB, Kirby–Bauer (mm); S, sensitive.

In brief, the clinical course progressed as follows: the patient initially presented with septic shock and abdominal distension, followed by diagnostic imaging confirming XGP and elevated intra-abdominal pressure. Emergency antimicrobial therapy and ultrasound-guided nephrostomy were performed for early source control. After hemodynamic stabilization in the intensive care unit, staged radical nephrectomy was undertaken. The patient subsequently recovered with gradual resolution of organ dysfunction and preserved renal function at follow-up.

## Discussion

### Xanthogranulomatous pyelonephritis: clinical features, diagnosis, and conventional management

XGP is a rare form of chronic pyelonephritis, characterized by kidney tissue destruction and the accumulation of foam cells. It often presents with nonspecific symptoms like fever, flank pain, and general discomfort, making it hard to diagnose. XGP is usually linked to UTIs, urinary blockages, and kidney stones, although cases without stones can occur, as seen in this patient with substance abuse. Although renal calculi are classically associated with XGP, an increasing number of non-lithogenic cases have been reported, suggesting alternative pathogenic pathways. Chronic methamphetamine abuse has been associated with immune dysregulation, including impaired T-cell–mediated immunity, altered macrophage activation, and increased susceptibility to persistent infections. Experimental and clinical studies have demonstrated that stimulant-induced immune dysfunction may promote chronic inflammatory responses and defective pathogen clearance. In this context, we hypothesize that substance-induced immune dysregulation may have contributed to the development of non-lithogenic XGP in this patient. Nevertheless, this association remains speculative and requires further validation through larger clinical cohorts and mechanistic studies. This proposed association should therefore be interpreted as a possible explanation rather than a confirmed pathogenic mechanism.

Diagnosis involves clinical, lab, and imaging findings. CT is the preferred imaging tool, showing enlarged kidneys, cortical atrophy, and the characteristic “bear paw sign” caused by dilated calyces filled with necrotic material^[^2^**]**^. In this case, CT confirmed the diagnosis.

Treatment includes broad-spectrum antibiotics for common uropathogens, such as *Proteus mirabilis, Escherichia coli*, and *Klebsiella pneumoniae*. Empiric antibiotic choices depend on local resistance patterns and the patient’s condition. Percutaneous nephrostomy is often used to drain pus and stabilize the patient. Definitive treatment is usually a radical nephrectomy, performed after infection control to prevent recurrence.

### XGP complicated by sepsis and abdominal compartment syndrome: rarity and management strategies

Sepsis and ACS as complications of XGP are very rare. Sepsis occurs when the infection spreads throughout the body, and ACS results from the enlarged kidney pressing on surrounding structures, along with systemic inflammation and fluid buildup^[^3,4^**]**^.

Risk factors for ACS in XGP include severe hydronephrosis, widespread kidney inflammation, and conditions like obesity or chronic kidney disease. Management of severe XGP with ACS necessitates a damage control approach, prioritizing rapid stabilization and delayed definitive repair^[^5,6^**]**^. Initial measures include aggressive fluid resuscitation, broad-spectrum antibiotics, and percutaneous drainage to reduce intra-abdominal pressure. Radical nephrectomy, while definitive, should be deferred until the patient is hemodynamically stable to minimize perioperative risks.

A limited number of cases of XGP complicated by sepsis have been reported in the literature, and reports describing concurrent abdominal compartment syndrome are exceedingly rare. Compared with previously published cases, the present report is distinguished by the simultaneous occurrence of septic shock and clinically significant intra-abdominal hypertension, the absence of renal calculi, and the successful application of a staged damage control strategy. These features emphasize both the diagnostic complexity and the therapeutic challenges of this unique clinical presentation.

### Multidisciplinary management of critically ill patients with XGP-induced septic shock

The treatment of critically ill patients with septic shock from XGP requires a team approach involving urologists, intensivists, infectious disease specialists, radiologists, and rehabilitation teams. Early involvement from these specialists ensures comprehensive care, from resuscitation and antibiotics to surgery and rehab. In this case, the patient’s recovery was due to early drainage, proper antibiotics, and staged surgery, with support from intensive care and rehabilitation. Multidisciplinary collaboration played a pivotal role in the successful management of this critically ill patient^[^7^]^. The infectious disease team guided empiric and targeted antimicrobial therapy, recommending cefoperazone/sulbactam, based on local antimicrobial resistance patterns and susceptibility testing results, which demonstrated β-lactam sensitivity of *Proteus mirabilis*. The critical care team closely monitored organ function and intra-abdominal pressure via bladder pressure measurements, adjusted fluid resuscitation strategies according to SOFA scores, and optimized ventilatory and hemodynamic support. The urology team performed ultrasound-guided percutaneous nephrostomy for early source control, followed by staged radical nephrectomy once hemodynamic stabilization was achieved. This coordinated, stepwise approach was essential in preventing further organ deterioration and achieving a favorable outcome.

### Implications for Artificial Intelligence-driven research in rare critical diseases

Recent advances in artificial intelligence (AI) have increasingly reshaped modern medical research and clinical decision-making, particularly in the domains of medical image analysis, multimodal data integration, and dynamic risk prediction. These developments provide new opportunities to address the inherent limitations of experience-driven clinical models, especially in the diagnosis and management of rare and life-threatening conditions.

Breakthroughs in AI-driven biomedical research, such as protein structure prediction and advanced clinical imaging analytics, have demonstrated the transformative potential of data-driven approaches in uncovering complex biological mechanisms and supporting personalized diagnosis and treatment strategies^[^8,9^]^. These technologies highlight how AI can move beyond auxiliary roles and contribute to the reconstruction of diagnostic and therapeutic logic in modern medicine.

In the context of the present case, AI-assisted image recognition algorithms could potentially be developed to identify characteristic radiological features such as the “bear paw sign” on abdominal computed tomography scans, thereby facilitating earlier recognition of XGP, particularly in atypical or non-lithogenic presentations. Furthermore, machine learning-based predictive models integrating clinical, laboratory, and hemodynamic parameters may help stratify the risk of sepsis-associated abdominal compartment syndrome and support timely decision-making regarding damage control strategies.

Although this report represents a single case, it highlights critical diagnostic and therapeutic challenges – such as delayed identification of non-calculous XGP, the unpredictable progression of sepsis-related intra-abdominal hypertension, and the time-sensitive coordination required for multidisciplinary intervention – that are well suited for future AI-driven, multi-center collaborative research. By transforming fragmented critical care experiences into quantifiable and reusable decision models, artificial intelligence may play a pivotal role in optimizing the management of rare but catastrophic complications and advancing precision medicine in critical care.

## Patient perspective

The patient expressed sincere gratitude for the intervention that ultimately saved her life. While the diagnosis and the decision to undergo nephrectomy were challenging, she appreciated the exceptional care provided by the medical team. She acknowledged that, although the experience was difficult, the coordinated approach and timely surgical intervention played a crucial role in her recovery.

The patient is committed to regular follow-up visits and renal function assessments to ensure her long-term health and monitor for any future issues. She recognizes the importance of continued care to maintain her well-being.

## Limitations

This study has several limitations inherent to the nature of a single-center retrospective case report. First, the findings are based on an individual clinical observation, which limits the generalizability of the conclusions to broader patient populations. Although the presented case highlights a rare and severe clinical scenario, causal inferences regarding pathophysiological mechanisms and treatment strategies cannot be definitively established.

Second, retrospective case reports are subject to selection bias and incomplete control of confounding variables. Certain clinical decisions, including the timing of interventions and antimicrobial selection, were influenced by real-time clinical judgment and institutional practice patterns rather than predefined protocols.

Third, while this report provides detailed clinical, radiological, and pathological documentation, it lacks comparative data or long-term follow-up across multiple cases. As such, the ability to assess prognostic factors or to systematically evaluate alternative management strategies is limited.

Nevertheless, despite these limitations, case reports remain a valuable tool for identifying rare disease presentations, generating hypotheses, and sharing practical clinical insights that may inform future observational studies or prospective research.

## Conclusion

This case of XGP complicated by sepsis and abdominal compartment syndrome highlights the severity and rarity of this condition. The successful outcome was achieved through a combination of aggressive initial management, damage control surgery, and multidisciplinary collaboration. This case underscores the importance of early recognition, appropriate antibiotic selection, and staged surgical intervention in managing severe XGP. Future studies should focus on identifying risk factors for ACS in XGP and optimizing multidisciplinary management strategies to improve outcomes in critically ill patients. The case has been reported in line with the SCARE 2025 criteria^[^10^]^.

## Data Availability

The original contributions presented in the study are included in the article/Supplementary Material. Further inquiries can be directed to the corresponding authors.

## Research registration unique identifying number (UIN)

As this study represents a retrospective case report, prospective research registration was not applicable.
